# SLIM: an alternative Web interface for MEDLINE/PubMed searches – a preliminary study

**DOI:** 10.1186/1472-6947-5-37

**Published:** 2005-12-01

**Authors:** Michael Muin, Paul Fontelo, Fang Liu, Michael Ackerman

**Affiliations:** 1 Office of High Performance Computing and Communications, National Library of Medicine, 8600 Rockville Pike, Bethesda, Maryland 20894, USA

## Abstract

**Background:**

With the rapid growth of medical information and the pervasiveness of the Internet, online search and retrieval systems have become indispensable tools in medicine. The progress of Web technologies can provide expert searching capabilities to non-expert information seekers. The objective of the project is to create an alternative search interface for MEDLINE/PubMed searches using JavaScript slider bars. SLIM, or Slider Interface for MEDLINE/PubMed searches, was developed with PHP and JavaScript. Interactive slider bars in the search form controlled search parameters such as limits, filters and MeSH terminologies. Connections to PubMed were done using the Entrez Programming Utilities (E-Utilities). Custom scripts were created to mimic the automatic term mapping process of Entrez. Page generation times for both local and remote connections were recorded.

**Results:**

Alpha testing by developers showed SLIM to be functionally stable. Page generation times to simulate loading times were recorded the first week of alpha and beta testing. Average page generation times for the index page, previews and searches were 2.94 milliseconds, 0.63 seconds and 3.84 seconds, respectively. Eighteen physicians from the US, Australia and the Philippines participated in the beta testing and provided feedback through an online survey. Most users found the search interface user-friendly and easy to use. Information on MeSH terms and the ability to instantly hide and display abstracts were identified as distinctive features.

**Conclusion:**

SLIM can be an interactive time-saving tool for online medical literature research that improves user control and capability to instantly refine and refocus search strategies. With continued development and by integrating search limits, methodology filters, MeSH terms and levels of evidence, SLIM may be useful in the practice of evidence-based medicine.

## Background

There is unprecedented growth of medical information. PubMed, a service of the National Library of Medicine, includes over 15 million citations of biomedical articles [1]. MEDLINE, the largest component of PubMed, covers over 4,800 journals. Searching for relevant and updated information in MEDLINE/PubMed can be challenging. Keyword searches without search limits and filters may retrieve thousands of citations.

Several studies have demonstrated that the number of physicians using the Internet is increasing [2-4]. De Groote and Dorsch confirmed that a large percentage of users in academic health sciences prefer online resources over print, and many choose to access these online resources remotely [5]. Convenience and availability of full text journals were important factors in selecting online resources to use. These trends suggest that widely available Web technologies that enhance browsing capabilities may improve MEDLINE/PubMed search experiences.

Many MEDLINE/PubMed searches start with single-word terms or free-text phrases. Wildemuth and Moore [6] noted that this search technique is prone to syntactical or typographical errors but can be improved with the use of an online thesaurus and the inclusion of synonyms in the search concepts. Entrez, the integrated, text-based search and retrieval system used for PubMed [7], addressed this issue by integrating Medical Subject Headings (MeSH) in the search process. MeSH is a controlled vocabulary thesaurus of the National Library of Medicine available online through the MeSH Browser [8].

The practice of evidence-based medicine encourages the selection of appropriate current evidence through effective literature searches. The process includes the refinement of the search strategy and evaluation of search results. This may require familiarity with PubMed search limits, clinical study filters and MeSH terms. Gallagher, Allen and Wyer [9] stated that "MeSH headings make the searcher less dependent on the words the author of an article chooses to use in the abstract or title and allow the searcher to rely more on the actual content of the article." The use of MeSH headings, MeSH subheadings and text words to refine and redirect MEDLINE searches can make the search process more effective.

The goal of the project was to develop a Web-based application with an alternative search interface using slider controllers to implement search limits, methodology filters and MeSH terminologies. The intentions were to enhance user interaction with the MEDLINE/PubMed database, allow immediate refinement and redirection of search strategies and provide options to easily use the MeSH thesaurus.

## Implementation

### Interface development and design

SLIM, or Slider Interface for MEDLINE/PubMed searches, is a Web-based application accessible through the Internet with a Web browser. The interface has three main components: the search form, the information box and the search results.

**Figure 1:**
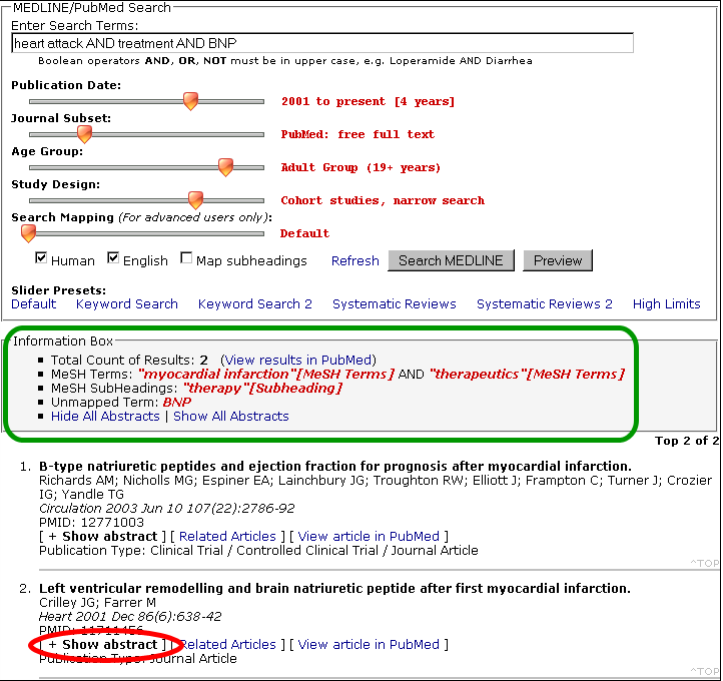
SLIM interface showing its three main components: search form, information box and search results. Green box: Information box. Red ellipse: Text link to display abstract

The search form (Figure 1) is the constant component in all views of the application and the only component initially loaded. It contains a text box for the search terms along with five slider controls. The JavaScript slider bars move between arbitrary values of 0 to 100 with mouse clicks or keyboard strokes. The default setting for all slider controls is zero, i.e. no limits or filters. Checkboxes are available for limits not appropriate for slider controls, such as Human studies and English language limits.

SLIM allows a preview of the number of results through the information box (Figure 1.) The information box is displayed below the search form for both previews and full searches. It also provides additional search information such as mapped MeSH Terms, mapped Subheadings and unmapped terms.

**Figure 2:**
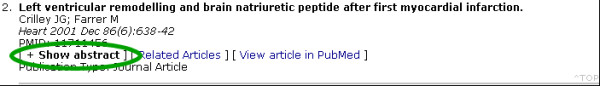
Citation view with abstract hidden. Green ellipse: Text link to show abstract

**Figure 3:**
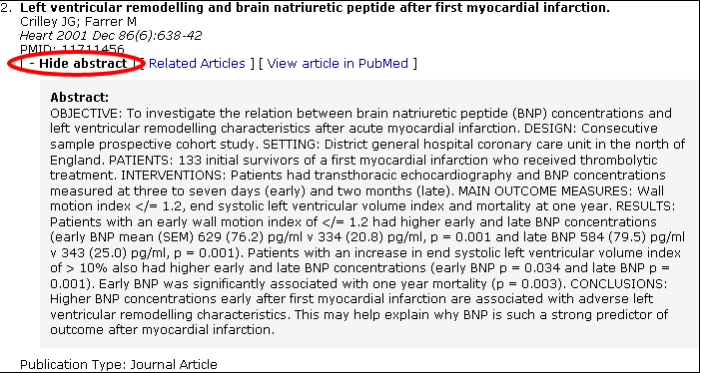
Citation view with abstract displayed. Red ellipse: Text link to hide abstract

When a full search is done, the results are presented below the information box. Each citation contains the article title, authors, journal name, volume, issue, pages and publication types. Abstracts, if available, are loaded on the page embedded with JavaScript functions that allow the abstracts to be hidden and displayed instantly with the click of a mouse (Figure 2 and 3). Abstracts are hidden by default. Links to related articles and PubMed are also provided.

### Limits and filters

SLIM follows the search and syntax rules of PubMed. Limits and filters are implemented by appending phrases and search tags to the search terms before querying the MEDLINE/PubMed database through the Entrez Programming Utilities (E-Utilities) [10]. The slider bars represent step implementations of common limits and filters in PubMed. Each slider bar holds values from 0 to 100, which are divided into subsets according to the number of options in that slider bar. When the slider is moved, the value where it stops is matched against the range of numbers assigned to a specific limit or filter. This scheme allows flexibility in designing the slider bar algorithms. Any changes (adding in or taking out filters) result in minor adjustments in the range of numbers in each subset.

The first three slider bars control search limits. These limits are Publication Date, Journal Subset and Age Groups. The three limits are modified versions of the Limits tab of PubMed. For the publication date, the slider has 11 steps and allows the user to choose different date ranges from 10 years back to the current year. The second slider contains 11 journal subset filters. Users have the option to search within the PubMed database, the MEDLINE subset [11] or the Core Clinical Journals [12]. Within each subset, users can limit results to availability of abstracts, full text or free full text. Full text articles provide links to publishers and may require subscription. Table 1 lists the journal subset descriptions and the corresponding PubMed limits. The age group slider is a reordered version of the age group drop-down menu in PubMed. It has 13 options starting with "Newborn" and ending with "80 and over". Table 2 lists the age group descriptions and the corresponding PubMed limits.

**Table 1:** Journal subset slider bar limit descriptions and corresponding PubMed filters

**Slider Value**	**Limit Description**	**PubMed Filter**
0	Default (All PubMed)	No filter
1–5	PubMed: with abstracts only	AND hasabstract
6–15	PubMed: full text	AND full text[sb]
16–25	PubMed: free full text	AND free full text[sb]
26–35	MEDLINE (4800+ journals)	AND medline[sb]
36–45	MEDLINE: with abstracts only	AND medline[sb] AND hasabstract
46–55	MEDLINE: full text	AND medline[sb] AND full text[sb]
56–65	MEDLINE: free full text	AND medline[sb] AND free full text[sb]
66–75	Core Clinical (120 journals)	AND jsubsetaim
76–85	Core Clinical: with abstracts only	AND jsubsetaim AND hasabstract
86–95	Core Clinical: full text	AND jsubsetaim AND full text[sb]
95–100	Core Clinical: free full text	AND jsubsetaim AND free full text[sb]

**Table 2:** Age group slider bar limit description and corresponding PubMed filters

**Slider Value**	**Limit Description**	**PubMed Filter**
0	Default	No age limits
1–8	Newborn (Birth – 1 month)	"infant, newborn"[MeSH Terms]
9–16	Infant (1–23 months)	"infant"[MeSH Terms:noexp]
17–24	Infant Group (Birth-23 months)	"infant"[MeSH Terms]
25–32	Preschool Child (2–5 years)	"child, preschool"[MeSH Terms]
33–40	Child (6–12 years)	"child"[MeSH Terms:noexp]
41–48	Adolescent (13–18 years)	"adolescent"[MeSH Terms]
49–56	Child Group (0–18 years)	("infant"[MeSH Terms] OR "child"[MeSH Terms] OR "adolescent"[MeSH Terms])
57–64	Adult (19–44 years)	"adult"[MeSH Terms:noexp]
65–72	Middle Aged (45–64 years)	"middle aged"[MeSH Terms]
73–80	Middle Aged + Aged (45+ years)	("middle aged"[MeSH Terms] OR "aged"[MeSH Terms])
81–88	Adult Group (19+ years)	"adult"[MeSH Terms]
89–96	Aged (65+ years)	"aged"[MeSH Terms]
97–100	80 and over (80+ years)	"aged, 80 and over"[MeSH Terms]

The fourth slider bar is a study design filter based on publication types, study designs or citation subsets. Although each filter within the slider functions independently, the slider values are listed according to the hierarchy of levels of evidence [13,14]. Three study designs, case-control studies, cohort studies and randomized controlled trials, are further divided into broad or narrow searches, giving a total of 9 slider levels. Research methodology filters for therapy [15] were adopted for randomized control trials. Other filters from the Clinical Studies Categories [15], e.g. diagnosis and prognosis, were not incorporated and will be evaluated for future versions of the application. The systematic reviews subset of PubMed was used unmodified for systematic review searches [16]. Table 3 lists the study design levels and the corresponding PubMed filters used for the slider control.

**Table 3:** Study design slider bar limit descriptions and corresponding PubMed filters

**Slider Value**	**Limit Description**	**PubMed Filter**
0	Default	No filters
1–12	Case Reports	AND ("case reports"[Publication Type] OR (case report[TIAB] OR case reported[TIAB] OR case reporting[TIAB] OR case reports[TIAB]))
13–24	Cross-sectional Surveys	AND ("cross-sectional studies"[TIAB] NOT Medline[SB]) OR "cross-sectional studies"[MeSH Terms] OR (cross [TIAB] AND sectional [TIAB])
25–36	Case-control studies, broad search	AND ((case control stud*[TIAB] NOT Medline[SB]) OR "case-control studies"[MeSH Terms] OR case-control stud* [TIAB])
37–48	Case-control studies, narrow search	AND (case control stud*[TIAB] OR "case-control studies"[MeSH Terms:noexp] OR "case-control studies"[MAJR])
49–60	Cohort Studies, broad search	AND ("cohort studies"[MeSH Terms] OR (cohort[TIAB] AND stud* [TIAB]))
61–72	Cohort Studies, narrow search	AND ("cohort studies"[MeSH Terms:noexp] OR "cohort studies"[MAJR])
73–84	Randomized Controlled Trials, broad search [15]	AND ((clinical[Title/Abstract] AND trial[Title/Abstract]) OR clinical trials[MeSH Terms] OR clinical trial[Publication Type] OR random*[Title/Abstract] OR random allocation[MeSH Terms] OR therapeutic use[MeSH Subheading])
85–96	Randomized Controlled Trials, narrow search [15]	AND (randomized controlled trial[Publication Type] OR (randomized[Title/Abstract] AND controlled[Title/Abstract] AND trial[Title/Abstract]))
97–100	Systematic Reviews [16]	AND systematic[sb]

Search mapping is a slider bar designed for intermediate to advanced users of PubMed. The filters use search tags and MeSH term operations to modify the search query. Search tags and MeSH term operations are short words or phrases enclosed in square brackets and appended to the keywords to refine search strategies. Initial modification substitutes the "[Text Word]" search tag with the "[TIAB]" search tag that redirects the search to keywords in the Title or Abstract. Subsequent levels in the slider involve adding mapped MeSH terms as filters, with options to search MeSH terms as major topics, or exclude MeSH terms below the current term in the MeSH tree. Table 4 describes the search mapping algorithm.

**Table 4:** Search mapping slider bar limit description and corresponding PubMed filter algorithm

**Slider Value**	**Limit Description**	**PubMed Filter Algorithm**
0	Default	No change in details
1–14	Mapped keywords searched in Title and Abstract	Keyword[Text Word] → Keyword[TIAB]
15–29	**ANY **Mapped MeSH Terms required	TIABDetails + **AND **(MH **OR **MH)
30–44	**ANY **Mapped MeSH Terms required but with no tree explosion	TIABDetails + **AND **(MH:noexp **OR **MH:noexp)
45–59	**ALL **Mapped MeSH Terms required	TIABDetails + **AND **(MH **AND **MH)
60–74	**ANY **Mapped MeSH Terms should be MAJOR topic	TIABDetails + **AND **(MH[MAJR] **OR **MH[MAJR])
75–89	**ALL **Mapped MeSH Terms required but with no tree explosion	TIABDetails + **AND **(MH:noexp **AND **MH:noexp)
90–100	**ALL **Mapped MeSH Terms should be MAJOR topic	TIABDetails + **AND **(MH[MAJR] **AND **MH[MAJR])

MeSH subheadings are topical qualifiers that describe a particular aspect of a subject such as etiology or therapeutic use. Users have the option to require these qualifiers through a checkbox. The MeSH subheadings are grouped and appended as a filter to the search query. This feature is closely linked with the search mapping algorithm. The subheading filter is only added if the search mapping algorithm requires MeSH terms, i.e. second level and above.

### System architecture and development

SLIM was written in PHP and developed on an Apache 2.0.52 server running PHP 4.3.10. The PHP scripts generate a HyperText Markup Language (HTML) and JavaScript search form. JavaScript provides most of the functionality of the search form and search results. Free and open source JavaScript codes were downloaded from the Internet for the slider controls [17,18]. Customized JavaScript codes were written to enhance the usability of the form and slider controls by providing pre-defined limit combinations, tool tips and slider labels.

The application connects to the MEDLINE/PubMed database using tools from Entrez Programming Utilities (E-Utilities) [10]. The ESearch tool searches and retrieves primary IDs and term translations. The EFetch tool retrieves records from a list of one or more primary IDs. The E-Utilities server generates remote XML documents for both processes. Custom PHP scripts were written to parse the XML files. SLIM sends two successive passes through the ESearch tool and one final pass through the EFetch tool when the search form is submitted.

Automatic term mapping is the process where terms entered in the PubMed query box without a search tag are matched against the Medical Subject Headings translation table, the journals translation table, the full author translation table and an author index [19]. To optimize modifications done by SLIM on the search terms, it was essential to emulate the mapping and translation algorithms of Entrez PubMed. The first ESearch pass was designed to mimic this process. By sending unmodified search terms to the E-Utilities server and retrieving the translation stack from the XML document, a custom PHP function was able to build the detailed search query from the parsed XML elements. The goal was to capture the process found in the Details tab of PubMed. Using the translation stack, mapped MeSH terms and subheadings were identified and recorded. Terms tagged as "All Fields" were identified as unmapped terms.

Depending on slider bar and search form input, the detailed search query built on the first ESearch pass is modified by appending user-defined limits and study design filters, or by converting search tags. The modified query is processed once again through the ESearch tool to get the final list of PubMed IDs (PMIDs) from the second XML document. The ID list is sent to the EFetch tool to retrieve and display the details of the first 200 citations.

### Performance and usability testing

To simulate performance testing, benchmarking timer functions were embedded in the PHP scripts to measure page loading and search times. All values generated during the alpha-testing with developers and beta-testing with users were recorded in a MySQL database for data analysis.

An online survey form was created to gather preliminary user opinion on stability and usability of the application. All seven questions used Likert scales to record answers. A call for participation in the usability testing was made on a mailing list of an international group of practicing physicians. Users were asked to use the system as a replacement for their regular PubMed search engine for two weeks. Comments and discussion on the application were encouraged.

## Results

Initial qualitative testing by application developers demonstrated that SLIM is functional and stable. Occasional XML file connection problems occurred, but rarely. These were attributed to technical issues in the E-Utilities server and were quickly resolved by resubmitting the search form. The application was tested for compatibility with multiple browsers and worked in default installations of Internet Explorer 6.0, Mozilla Firefox 1.06, Opera 8.02 and Safari 1.2.

During performance testing, the index page was loaded 216 times with a mean page generation time of 2.94 milliseconds and standard deviation of 3.20 milliseconds. Search previews were processed 65 times with a mean page generation time of 0.63 seconds and standard deviation of 0.78 seconds. Complete searches with display of results were done 142 times with a mean page generation time of 3.84 seconds and standard deviation of 6.28 seconds. Table 5 gives a summary of the page generation time tests including standard deviations and quartiles.

**Table 5:** Summary of Page Generation Times

	**Index Page**	**Preview Page**	**Search Page**
Number of Loading Tests	216	65	142
Mean	2.94 ms	0.63 s	3.84 s
Standard deviation	3.20 ms	0.78 s	6.28 s
Minimum value	2.38 ms	0.11 s	0.00 s
25th percentile	2.48 ms	0.30 s	0.52 s
Median	2.53 ms	0.41 s	1.12 s
75th percentile	2.61 ms	0.62 s	3.61 s
Maximum value	47.85 ms	5.54 s	35.04 s

**ms – **milliseconds

**s – **seconds

Eighteen physicians from the US, Australia and the Philippines participated in the beta-testing phase of the application and provided performance and usability feedback through an online survey. The mode was used as measure of central tendency as recommended by experts because Likert scales fall within the ordinal level of measurement [20,21]. Table 6 lists the tabulated answers and mode score for each statement in the user survey. Seven users agreed that the slider interface is more user-friendly than traditional search interfaces. Nine users found the Web-based application stable, while nine users strongly agreed that speed was acceptable. Eight users thought that the interface was easy to use. Seven physicians strongly agreed that similar interactive features are desirable in other MEDLINE searches. Six strongly believed that seeing mapped MeSH terms and unmapped keywords in their searches is a useful feature. Nine strongly preferred the option to hide and display the abstracts.

**Table 6:** Tabulation of answers and mode scores from user survey (n = 18)

	**Statements**	**Answers/Total Respondents**					**Mode Score**
			
		**1 SD**	**2 D**	**3 N**	**4 A**	**5 SA**	
1	The new interface with slider controls is more user-friendly than traditional search interfaces.	1/18	3/18	4/18	**7**/18	3/18	**4**
2	The Web-based application is stable.	0/18	0/18	5/18	**9**/18	4/18	**4**
3	The speed of search and results display is acceptable.	0/18	0/18	1/18	8/18	**9**/18	**5**
4	The new interface is easy-to-use with minimal confusion on how controls work.	0/18	2/18	5/18	**8**/18	3/18	**4**
5	I'd like to see similar interactive features in other MEDLINE search engines.	1/18	1/18	5/18	4/18	**7**/18	**5**
6	Seeing mapped MeSH terms and unmapped keywords in the information box is a useful feature.	0/18	3/18	4/18	5/18	**6**/18	**5**
7	I like the feature where I can hide and display abstracts.	0/18	1/18	3/18	5/18	**9**/18	**5**

**1 – **Strongly disagree (**SD**)

**2 – **Disagree (**D**)

**3 – **Neutral (**N**)

**4 – **Agree (**A**)

**5 – **Strongly agree (**SA**)

## Discussion

### Search interface design

Research in data mining, natural language processing and methodology filters move towards developing backend algorithms and protocols for online information search and retrieval systems. Despite all these advances, users continue to use default settings in their PubMed searches. This is a preliminary study of an ongoing project to improve search interface usability by enhancing user interaction with advanced features of PubMed.

Limits and filters were chosen based on adaptability to structured series of implementations. Publication date and age groups were obvious choices. The journal subset slider narrowed down the search pool by combining subsets in PubMed with selected filters for availability of text. The hierarchy of levels of evidence provided a structured implementation for publication types and clinical study categories. The search mapping feature increased user involvement with MeSH operations. The project continues to study most of the limits and filters available in PubMed for integration in future versions of the application.

The information box was created as an educational tool for PubMed users and researchers by providing another level of feedback. It is nothing more than a short report on the term mapping and translation algorithms of Entrez PubMed. Users are informed of the MeSH term or MeSH subheading equivalents of their search terms. Unmapped terms can prompt users to modify specific keywords in their search.

### Usability survey

Users who participated in the online survey were practicing physicians interested or involved in Medical Informatics. All were frequent users of PubMed and categorized themselves as intermediate to advanced users. The familiarity with the current Entrez PubMed interface often accounts for the negative responses in the online survey. Although most user comments were positive, one user stated that advanced users of PubMed might feel more comfortable with the consistent interface of Entrez PubMed, whereas slider controls might be more useful for novice or non-expert users. One user suggested that slider labels be more descriptive. In response, some label texts were expanded to include descriptions of the slider settings. A tutorial page was also suggested.

### Limitations of the study

The study has several limitations. First, the search interface omits several limits existing in the PubMed limits page like subsets and non-English languages. The developers continue to look into integrating currently available filters in newer versions of the application. Second, only eighteen physicians interested in Medical Informatics projects participated in the beta-testing phase. A broad mix of PubMed researchers is a target for future usability studies. Third, the retrieval performance to measure sensitivity and specificity of the methodology filters was not tested. Review of Clinical Study Category filters not incorporated in the application, e.g. diagnosis and prognosis, is in process. Ongoing efforts to improve the application include evaluating search algorithms for accuracy and precision and adopting validated PubMed filters from previous published studies. The developers continue to monitor advancements in Web technology and add new interactive features in the application.

The goal of the project was to create a PubMed search application that allows users of all levels to easily go beyond basic keyword searches and move towards evidence-based principles. This is consistent with the practice of evidence-based medicine which advocates the formulation of effective search strategies to find current evidence. With increasing access to the Internet, online bibliographic databases have become important real-time resources for current evidence at the point-of-care. Although tools to control search parameters are available, these often require advanced familiarity with the search interface elusive to beginners. By exploring progressive Web technologies and creating an interactive search interface, the application may prove valuable in bridging the gap between expert and non-expert users of PubMed.

## Conclusion

The Web-based application offers an alternative search interface to facilitate MEDLINE/PubMed searches. JavaScript slider bars control search limits, add filters or modify search terms with ease. Textual link controls can hide or display abstracts with a mouse. Initial qualitative testing and user feedback were positive which reinforced the approach of enhancing user interaction to improve online research. SLIM can be an interactive time-saving tool for online medical literature research that improves user control and capability to instantly refocus search strategies. With continued development and by integrating search limits, methodology filters, MeSH terms and levels of evidence, SLIM may be useful in the practice of evidence-based medicine.

## Availability and requirements

**Project name**: SLIM (Slider Interface for MEDLINE/PubMed searches)

**Project home page**: 

**Operating systems**: Platform independent

**Programming language**: PHP, JavaScript

**Other requirements**: JavaScript-enabled browsers, e.g. Fire Fox 1.0 or higher, IE 5 or higher

**License**: Free, anyone may use the service

**Any restrictions to use by non-academics**: None

## List of abbreviations

SLIM: Slider Interface for MEDLINE/PubMed searches

MeSH: Medical Subject Headings

PHP: PHP: Hypertext Preprocessor

HTML: HyperText Markup Language

XML: Extensible Markup Language

PMID: PubMed ID

## Competing interests

The author(s) declare that they have no competing interests.

## Authors' contributions

MM conceived of the study, designed the interface, developed filters and algorithms, wrote the PHP codes, organized the usability testing and drafted the manuscript. PF assisted in the study and interface design, participated in usability testing, developed filters and algorithms and reviewed the initial drafts of the manuscript. FL wrote the custom JavaScript codes and assisted in design of the interface. MA participated in the study design, evaluation study and gave final approval of the version to be published. All authors read and approved the final manuscript.

## Pre-publication history

The pre-publication history for this paper can be accessed here:


